# Design and Physicochemical Characterization of Hybrid PLGA–Curcumin/Carbon Dot Nanoparticles for Sustained Galantamine Release: A Proof-of-Concept Study

**DOI:** 10.3390/biom16010176

**Published:** 2026-01-21

**Authors:** Christina Samiotaki, Stavroula Nanaki, Rizos Evangelos Bikiaris, Evi Christodoulou, George Z. Kyzas, Panagiotis Barmpalexis, Dimitrios N. Bikiaris

**Affiliations:** 1 Laboratory of Polymer Chemistry and Technology, Department of Chemistry, Aristotle University of Thessaloniki, 541 24 Thessaloniki, Greece; samiotaki@chem.auth.gr (C.S.); sgnanaki@chem.auth.gr (S.N.); evicius@gmail.com (E.C.); 2 Hephaestus Laboratory, School of Chemistry, Faculty of Sciences, Democritus University of Thrace, 654 04 Kavala, Greece; rizosbikiaris@gmail.com (R.E.B.); kyzas@chem.duth.gr (G.Z.K.); 3 Laboratory of Pharmaceutical Technology, Department of Pharmacy, Faculty of Health Sciences, Aristotle University of Thessaloniki, 541 24 Thessaloniki, Greece; pbarmp@pharm.auth.gr

**Keywords:** galantamine, curcumin, poly (lactic-co-glycolic acid), carbon dots, intranasal delivery

## Abstract

The present study reports the design and physicochemical characterization of a hybrid nanoparticle system for the potential intranasal delivery of galantamine (GAL), aimed at improving its bioavailability. Carbon dots (CDs) were used to load GAL, enhancing its dissolution and stability, and were subsequently incorporated into a poly(lactic-co-glycolic acid)–curcumin (PLGA–Cur) conjugate matrix. The successful formation of the PLGA-Cur conjugate was verified via ^1^H-NMR and FTIR spectroscopy, while the loading of GAL and its physical state in the CDs was assessed via FTIR and pXRD, respectively. The resulting GAL-CD/PLGA–Cur nanoparticles were spherical, with particle sizes varying from 153.7 nm to 256.3 nm, a uniform morphology and a narrow size distribution. In vitro release studies demonstrated a multi-phase sustained release pattern extending up to 12 days. Spectroscopic and thermal analyses confirmed successful conjugation and molecular interactions between GAL and the carrier matrix. This proof-of-concept hybrid system demonstrates promising controlled, multi-phase sustained galantamine release in vitro, highlighting the role of curcumin conjugation in modulating polymer structure and release kinetics and providing a foundation for future biological evaluation.

## 1. Introduction

Galantamine (GAL) stands as a pivotal therapeutic agent against neurodegenerative disorders, acting as a selective acetylcholinesterase (AChE) inhibitor. Its primary mode of action involves enhancement of cholinergic neurotransmission by inhibiting acetylcholine breakdown within the synaptic junctions [1,2]. In general, GAL is an active pharmaceutical ingredient (API) used for the treatment of Alzheimer’s disease (AD) by increasing the levels of specific natural compounds in the brain, that are essential for memory and cognition [3,4,5]. Current clinical practice for this API relies mostly on its oral administration, a route that, despite its widespread acceptance, is related to several stomach and bowel adverse events, such as nausea, vomiting, and diarrhea [6]. These drawbacks lead to treatment discontinuation in certain patients, while challenges arising from GAL’s difficulties to pass through the blood–brain barrier (BBB), rendering its oral administration less effective [7].

Within this context, creating novel drug delivery systems for GAL by selecting different administration routes, becomes crucial. A particularly promising alternative, ideal for treating AD patients with AChE inhibitors like GAL, is the intranasal (IN) route [8,9]. Recent reviews have emphasized the growing interest in developing multifunctional or hybrid galantamine derivatives and nanoformulations targeting multiple pathogenic pathways of Alzheimer’s disease, including cholinergic deficit, oxidative stress, and amyloid aggregation. Such approaches combine neuroprotective, antioxidant, and enzyme-inhibitory functions within a single platform, offering enhanced therapeutic promise compared to conventional monotherapies [10]. Generally, IN delivery is an uncomplicated and user-friendly way to circumvent the BBB, allowing direct medication delivery to the brain [11,12,13,14,15]. Its numerous benefits include a swifter drug action that can sidestep the first-pass metabolism, minimized side effects compared to systemic administration, precise drug targeting, and an easy and patient-preferred administration method [16,17,18]. Yet, despite these advantages, there is much more to explore and understand about the use of IN in GAL’s administration and its effectiveness in AD therapy.

Li et al. were the first to introduce the concept of IN delivery for GAL [19]. They used an innovative flexible liposomal system, which displayed a marked enhancement in GAL’s bioavailability. This system also demonstrated a commendable safety record without any toxic effects. Yet, despite the significant potential of liposomes, concerns about their stability and other practical challenges (such as their bio-adhesion) shifted focus towards alternative formulation approaches. This led to the evaluation of different methods to achieve effective IN delivery of GAL, such as the use of a nano-formulation that combined a cationic polymeric substance, chitosan (CS) [20], or the use of a new GAL prodrug variant [21]. Both studies yielded encouraging results concerning GAL’s safety and performance. In a subsequent research paper, the use of GLA-CS nanoparticles (NPs) was proved to diminish the AChE concentrations in the brain, while amplifying cholinergic activity [22]. Also, in a recent study coming from our research group the use of thiolized CS conjugates (with or without the use of carbon dots, CDs) was evaluated as a promising nano carrier system for the IN delivery of GAL [23]. Findings revealed that incorporating L-cysteine-modified CS with CDs produced a drug dispersion with enhanced thermodynamic stability and achieved a zero-order release profile. Although chitosan and its modified derivatives exhibit several favorable characteristics—such as natural origin, positive surface charge promoting cell adhesion, and strong mucoadhesive properties. Refs. [24,25,26], report that the specific requirements of Alzheimer’s disease treatment and galantamine delivery to the brain may necessitate alternative polymeric carriers. In this regard, biodegradable aliphatic polyesters, including poly(lactic acid) (PLA) and poly(lactic-co-glycolic acid) (PLGA), have been extensively explored as attractive options for developing hybrid nanoparticle-based delivery systems due to their biodegradability, biocompatibility, and mechanical strength [27,28]. Among these, PLGA and its modified derivatives often represent the most suitable choice for achieving the desired drug-release profile and stability. This choice, of course, largely depends on the specific drug’s properties (such GAL’s compatibility with the PLGA-based matrix/carrier), its mechanism of action, and the desired pharmacokinetics and pharmacodynamic profile.

Several innovative nanosystems have recently been investigated to enhance GAL brain delivery. For example, lipid–polymer hybrid nanoparticles have shown improved BBB penetration and sustained release kinetics [29], while thiolated chitosan and other mucoadhesive polymers demonstrated increased nasal retention and mucosal adhesion. Another strategy involved embedding GAL-loaded liposomes within polymeric scaffolds to achieve prolonged diffusion-controlled release and neuronal biocompatibility [30]. Similarly, in a previous work of our group, hierarchical porous carbon carriers integrated in PLLA/PLGA matrices enabled hippocampal targeting in vivo [31].

In this context, the present research study investigates the preparation of a hybrid GAL IN delivery systems, where the API is encapsulated in specialized CDs and then embedded into PLGA-curcumin (PLGA-Cur) modified NPs. Curcumin, the active compound in *Curcuma longa*, is a phytochemical renowned for its extensive range of biological activities, which include anti-cancer, anti-inflammatory, and notable antibacterial properties [32,33,34,35]. Beyond its protective effects, curcumin maintains the structural and functional integrity of blood vessels, energy-producing organelles (mitochondria), and connections between neurons (synapses), thereby helping to preserve cognitive function Curcumin also has natural fluorescent properties with stable wavelength emission. Its *β*-dicarbonyl structure has good reactivity which can form a variety of ring structures, which bind strongly to disease-related proteins, making it useful as a diagnostic tool for early detection [36]. In recent years, some curcumin-like fluorescent probes have been prepared and used to detect soluble/insoluble amyloids, intracranial reactive oxygen species, cysteine, and cancer cells. In addition, curcumin functions as a versatile release modulator primarily through controlling the release and activity of key cellular signaling molecules, such as growth factors, reactive oxygen species (ROS), and inflammatory cytokines. Concerning AD treatment, recent findings highlight curcumin’s potential in the management of AD, serving as both a diagnostic and therapeutic molecule [37]. It has revealed various potential mechanisms that could benefit AD patients, including promoting the removal of Aβ plaques through the stimulation of phagocytosis by immune cells [38]. It possesses potent antioxidant activity (superior to Vitamin E), reduces neuroinflammation and chelates harmful metal ions in the brain [39,40]. Despite these advances, few studies have combined PLGA with both curcumin and carbon-based nanostructures to achieve multifunctionality.

Therefore, the present study builds upon these earlier reports to propose a hybrid PLGA–Cur/CD formulation as a proof-of-concept system for potential intranasal GAL delivery. PLGA with a lactic-to-glycolic acid ratio of 65/35 was selected based on our previous work on intranasal galantamine delivery using carbon-based hybrid nanoparticles [31], where this copolymer composition exhibited favorable degradation kinetics and sustained drug release extending up to 10–12 days. Here, the proposed hybrid platform aims to co-deliver Cur and GAL (an approved AChE inhibitor), protect the drug, ensure a superior dissolution profile and improve its bioavailability. The present study is limited to physicochemical characterization and in vitro release, while biological validation and evaluation of the system’s therapeutic potential will follow [41]. In addition, the use of CDs as a carrier on which the GAL will be loaded before embedded into the PLGA-curcumin NPs, may facilitate a superior dissolution characteristics of GAL, due to CDs high surface-to-volume ratio, and may also offer an extra layer of protection to the drug, safeguarding it from potential degradative environments, and ensuring its stability until it reaches the target site.

## 2. Materials and Methods

### 2.1. Materials

Galantamine (GAL, purity ≥ 99%) (C_17_H_21_NO_3_ with a molecular mass of 287.35 g/mol) was kindly donated by Pharmathen S.A. (Athens, Greece). PLGA with 65/35 *w*/*w* lactic (L) to glycolic (G) acid ratio was kindly donated by Corbion (Amsterdam, The Netherlands). Curcumin (Cur) from *Curcuma longa* (Turmeric) in powder form, glutaric anhydride (95%, GA) and the 4-dimethylamino pyridine (DMAP, ≥99%), triethylamine ((Et)_3_N, ≥99.5%), tetrahydrofuran (THF, ≥99.9%), hydrochloric acid (HCl, 1 M), dichloromethane (DCM, ≥99.9%), methanol (MeOH, ≥99.9%), N, N′-dicyclohexylcarbodiimide (DCC, 99%) used for the synthesis of the new PLGA-Cur carrier were all purchased from Sigma-Aldrich Chemical Co. (Stainheim, Germany). All other chemicals were of analytical or pharmaceutical grade, while all solvents used in HPLC analysis were of HPLC grade.

### 2.2. Synthesis of the New PLGA-Cur Carrier

The synthesis of PLGA-Cur involved two stages. In the initial step, the curcumin molecule was bound to glycolic acid (Cur-GA), which was subsequently coupled to PLGA 65/35. For this, 1.55 g of Cur (≈4.21 mmol), 0.26 g of DMAP (≈2.13 mmol), 0.95 mL of triethylamine (≈6.8 mmol), and 80 mL of THF were combined in a 200 mL volumetric flask. Once dissolved, 0.51 g of glutaric anhydride (≈4.47 mmol) was introduced. The mixture’s temperature was raised to 55 °C and maintained for 17 h with continuous mechanical stirring, reflux, and a consistent nitrogen flow. Post-reaction, the THF was evaporated under vacuum. The resulting solid was then redissolved in 60 mL of ethyl alcohol and precipitated with 20 mL of a 1 M HCl solution, effectively eliminating the (Et)_3_N. Following the distillation of ethyl alcohol, Cur-GA was retrieved as a solid. Impurities were later removed using a silica gel-filled chromatographic column, employing a DCM:MeOH mixture at a 98:2 volume ratio as the eluent. For the second phase, the PLGA65/35 was bound to Cur-GA. Specifically, 1.1 g of PLGA 65/35 and 175 mg of Cur-GA, both dissolved in 50 mL of DCM, were placed in a round-bottom flask. To this, 52 mg of DMAP and 108 mg of DCC were added. This mixture was continuously stirred for 24 h before being precipitated in cold diethyl ether. The resultant yellow product underwent further purification in water using a transfiltration membrane to remove any unreacted curcumin.

### 2.3. Characterization of the New PLGA-Cur Carrier

#### 2.3.1. ^1^H NMR

The ^1^H-NMR spectra of the formulated systems were acquired in deuterated chloroform at room temperature using an Agilent 500 spectrometer (Agilent Technologies, Santa Clara, CA, USA). The acquisition parameters included 16 scans and a sweep width of 6 kHz.

#### 2.3.2. Fourier Transform Infrared (FTIR) Spectroscopy

FTIR spectra were obtained using an FTIR spectrometer (model FTIR-2000, Perkin Elmer, Dresden, Germany) with potassium bromide disks having a thickness of 500 μm. Spectra were recorded in the 4000 to 400 cm^−1^ range with a resolution of 2 cm^−1^ (20 co-added scans). The presented spectra underwent baseline correction and were converted to absorbance mode.

#### 2.3.3. Thermogravimetric Analysis (TGA)

Thermogravimetric analysis (TGA) was conducted using a Setsys 16/18 TG-DTA instrument (SETARAM Instrumentation, Caluire, France). Samples (10 ± 0.5 mg) were positioned in alumina crucibles, with an empty alumina pan as reference. The samples underwent heating from room temperature to 600 °C at a rate of 20 °C/min in a 50 mL/min N2 flow. Continuous records of sample temperature, weight, and heat flow were acquired. All measurements were carried out in triplicate.

#### 2.3.4. Differential Scanning Calorimetry (DSC)

DSC measurements were conducted using a Pyris Diamond DSC system (Perkin–Elmer, Dresden, Germany). System calibration was achieved using Indium and Zinc standards [42]. Precisely weighed samples (5 ± 0.1 mg) were enclosed in aluminum pans and subjected to heating in the range of 30 to 360 °C at a rate of 20 °C/min.

#### 2.3.5. Powder X-Ray Diffractometry (pXRD)

pXRD measurements were carried out utilizing a Rigaku XRD-diffractometer (Miniflex 600, Chalgrove, Oxford, UK) employing CuKα radiation for identifying crystalline phases (λ = 0.15405 nm for CuKα). Scans of all samples were conducted from 5° to 40° with a step scan of 0.05 deg and a scan speed of 1 deg/min.

### 2.4. Preparation of NPs

#### 2.4.1. CDs and GAL-Loaded CDs Fabrication

CDs synthesis followed a prior protocol [43], and their characterization has been provided in our previously published work [44]. Briefly, citric acid and urea (1/2 *w*/*w* ratio) dissolved in DMF underwent a solvothermal reaction at 160 °C for 5 h. The resulting product was precipitated in ethanol, collected by centrifugation (12,000 rpm, 30 min), and dispersed in ultrapure water via sonication. The process yielded spherical carbon nanostructures with an average diameter of approximately 4.5 nm. FTIR and XPS analyses confirmed the presence of characteristic surface functionalities, including C–O, C–N, and C=O bonds, contributing to their hydrophilicity and capacity for electrostatic and hydrogen-bonding interactions with galantamine and the polymer matrix. In addition, the CDs exhibited a mildly negative surface charge at neutral pH (ζ-potential in the range of −15 to −25 mV), attributed to the deprotonation of surface carboxyl and hydroxyl groups, as reported in our previous work and in similar citric-acid/urea-derived CD systems. This surface charge, together with the ultrasmall size and abundant polar functional groups, is known to promote stable drug adsorption, reduce aggregation, and modulate diffusion-controlled release from polymeric matrices.

For API loading, 50 mg of GAL dissolved in 100 mL methanol was mixed with a measured amount of CDs, yielding GAL-loaded CDs (GAL-CDs) via adsorption/surface complexation. After 24 h of magnetic stirring, the suspension underwent centrifugation (12,500 rpm, 20 min), and the precipitate was dried to eliminate excess methanol, yielding drug-loaded carbon dots (CDs) stored in a desiccator.

#### 2.4.2. Embedding GAL-CDs into the NPs

Polymeric nanoparticles loaded with GAL-CDs were fabricated using a modified solid-oil–water (s/o/w) double emulsification technique, as previously reported [45]. In this method, 150 mg of either pure PLGA or PLGA-Cur polymer dissolved in 5 mL of DCM, and 5 mg of GAL-CDs were incorporated into the polymeric solution. This mixture was dispersed using a probe sonicator (UP200H, Hielsher GmbH, Berlin, Germany), added to a 30 mL sodium cholate aqueous solution (0.1% *w*/*v*), and sonicated. After evaporation of dichloromethane, the resulting NPs were obtained by centrifugation, washed, freeze-dried, and stored at 4 °C. Neat GAL NPs were also prepared using the same procedure without CDs for comparison.

### 2.5. Characterization of NPs

#### 2.5.1. FTIR Spectroscopy, DSC and pXRD

The FTIR, DSC and pXRD of the prepared systems was evaluated using the same methodology as described previously.

#### 2.5.2. Particle Size and ζ-Potential via Dynamic Light Scattering (DLS)

Particle size distribution and zeta potential were assessed using DLS with a Nano-S Zetasizer (Malvern Instruments, Malvern, Worcestershire WR14 1XZ, UK). The self-optimization routine in the Zetasizer software (Version 3.30, Malvern Panalytical Ltd., Malvern, UK) was employed for all measurements, and ζ-potential was calculated based on the solutions theory. The samples were 100-fold diluted with a low ionic strength (2 mM) phosphate buffer at pH 7, and all measurements were conducted at 25 °C in triplicate. For batch-to-batch reproducibility assessment, three independent preparations per formulation were performed; each batch was measured in triplicate, and the batch mean was used to calculate the relative standard deviation (*RSD*) across batches:

$$\%\;RSD=\;\frac{SD\;of\;batch\;means}{grand\;mean}\;\times \;100$$

#### 2.5.3. Scanning Electron Microscopy (SEM)

Scanning Electron Microscope (SEM) images were acquired using a JEOL 2011 electron microscope (JEOL Ltd., Tokyo, Japan). Prior to imaging, samples of the prepared nanoparticles (NPs) were coated with carbon. The images were captured under the following operating conditions: accelerating voltage of 20 kV, probe current of 45 nA, and a counting time of 60 s.

#### 2.5.4. Drug Loading, Yield and Entrapment Efficiency (EE)

To determine drug loading, 1 mg of the prepared nanoparticles was dissolved in a mixture of dichloromethane and methanol in a 1:1 ratio. The resulting solution was then subjected to analysis using High-Performance Liquid Chromatography (HPLC). Drug loading, yield, and entrapment efficiency were computed using the following Equations:

Drug loading (%) = [(weight of drug in NPs)/(weight of NPs)] × 100(1)

Yield (%) = [(weight of NPs)/(initial weight of all materials)] × 100(2)

EE (%) = [(weight of drug in NPs)/(initial weight of drug)] × 100(3)

All measurements were performed in triplicate (*n* = 3), and data are expressed as mean ± standard deviation (SD).

#### 2.5.5. Dissolution Studies

For the in vitro release studies of GAL, a Distek Dissolution Apparatus (Evolution 2100C, North Brunswick Township, NJ, USA) equipped with an autosampler (Evolution 4300, North Brunswick Township, NJ, USA) was utilized following the USP I method (basket method). NPs weighing approximately 50 mg were enclosed in dialysis tubing cellulose membranes with a Molecular Weight Cut-Off (MWCO) of 12,000–14,000 (D9402-100FT, North Brunswick Township, NJ, USA) and positioned in the dissolution baskets. Dissolution analysis was carried out at 37 ± 1 °C with a rotation speed of 50 rpm, using 500 mL of phosphate-buffered saline (PBS) with a pH of 7.4 as the dissolution medium to maintain sink conditions and ensure drug stability during prolonged testing. Future studies will evaluate release behavior in nasal-simulated fluids (pH 5.5–6.5) to better represent intranasal conditions. Samples (2 mL) were withdrawn at specified time intervals, and GAL content was analyzed by HPLC. All measurements were performed in triplicate (*n* = 3).

For assessing the drug-release mechanism, the in vitro dissolution results were fitted to the following release kinetics models [46]:

Zero-order model: D_t_ = D_0_ + k_0_t (4)

First-order model: logD_t_ = logD_0_ + k_1_t/2.303 (5)

Higuchi square root model: D_t_ = D_0_ + k_H_t^1/2^ (6)

Hixon-Crowell model: D_t_^1/3^ = D_0_^1/3^ − k_HC_t (7)

Korsmeyer–Peppas model: D_t_/D_∞_ = D_0_ + k_P_t^n^ (8)

where D_t_ is the amount of drug released at time t, D_0_ is the initial amount of drug released, D_t_/D_∞_ is fraction of drug released at time t, k_0_ is the zero-order release constant, k_1_ is the first-order release constant, k_H_ is the Higuchi release constant, k_HC_ is the Hixson–Crowell release rate constant, k_P_ is the Peppas release constant, and *n* is the release exponent, respectively.

#### 2.5.6. HPLC Analysis for the Determination of GAL

GAL quantification was conducted using a Shimadzu Prominence HPLC system (LC-20AD, Tokyo, Japan). The analysis utilized a C18 column (CNW Technologies Athena, 120 Å, 5 μm, 250 mm × 4.6 mm, Tokyo, Japan) maintained at 25 °C. The mobile phase comprised a 10 mM aqueous solution of KH_2_PO_4_ with a pH of 3.5 as phase A and methanol as phase B, with a ratio of A/B 80/20 *v*/*v*. The flow rate was set at 1.0 mL/min, an injection volume of 10 μL, and GAL analysis was conducted at 235 nm.

#### 2.5.7. Statistical Analysis

All experimental measurements were performed in triplicate as technical replicates obtained from a single nanoparticle preparation per formulation. Results are presented as mean ± standard deviation (SD). The reported SD values reflect analytical and instrumental repeatability rather than batch-to-batch variability. Given that independent nanoparticle batches were not prepared for each formulation, inferential statistical analyses were not applied. Accordingly, the quantitative data are interpreted using descriptive statistics, and comparative statements are framed as observed trends in mean values rather than statistically validated differences.

For nanoparticle size measurements, three independent nanoparticle batches were prepared per formulation, and relative standard deviation (RSD) values were calculated. Dissolution kinetic model fitting was evaluated using the correlation coefficient (R^2^) statistical parameter.

## 3. Results and Discussion

### 3.1. Synthesis and Characterization of the New PLGA-Cur Carrier

**Figure 1:**
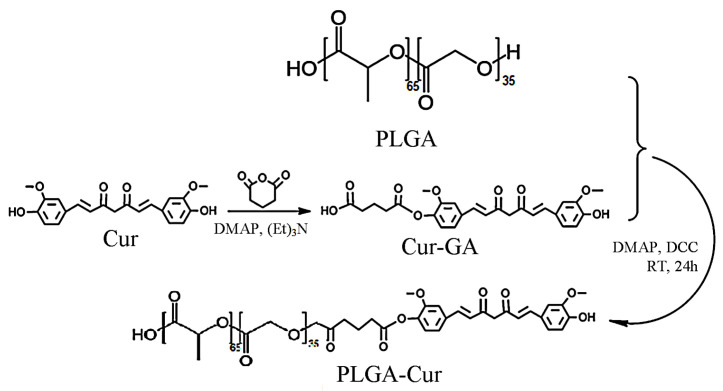
Reaction scheme for the preparation of a conjugated PLGA-Cur.

In this study, the synthesis of a novel conjugate involving the modification of PLGA with Cur was carried out via a two-step synthetic process, as illustrated in Figure 1. Following the reaction and removal of residual water through freeze-drying, the resulting dried conjugate appeared as an odorless powder with shades ranging from orange to yellow.

**Figure 2:**
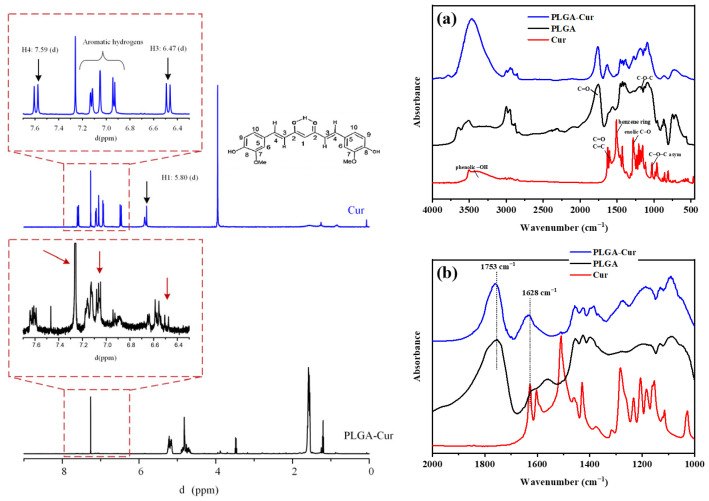
**Left**: ^1^H-NMR spectra of the neat Cur and the new conjugated PLGA-Cur. (**Right**): FTIR spectra from 4000 to 400 cm^−1^ (**a**) and 2000 to 1000 cm^−1^ (**b**) of the neat Cur the neat PLGA and the conjugated PLGA-Cur.

Figure 2 presents the ^1^H-NMR spectrum of pure Cur as well as its conjugate with PLGA (PLGA-Cur). For the pure Cur, the spectrum clearly depicts the signal for H-1 at δ 5.80 ppm as a doublet, while the H-3 signal appears at δ 6.47 ppm as a doublet. Furthermore, the signal for H-4 is noted at δ 7.590 ppm, and the signals for the aromatic hydrogens range from 6.90 to 7.15 ppm. These observations align with previously published data on the ^1^H NMR spectrum of Cur [47]. In the case of the pure PLGA spectrum (data not provided), several ^1^H-NMR peaks emerge those around ~1.6 ppm are associated with the repetitive methyl groups of lactic acid, and those at 5.2 ppm and 4.8 ppm correspond to the CH-CH3 from lactic acid and CH-H from glycolic acid, respectively. Notably, the intensity of the peak at 4.8 ppm serves as an indicator of the glycolic monomer within the copolymer, which can help ascertain the actual L/G ratio in the copolymer [48,49]. Upon examination of the NMR spectrum for the conjugated PLGA-Cur, noticeable shifts in the aromatic and methylene bridged protons of curcumin towards higher ppm values were observed compared to pure Cur. These shifts indicate the binding of the aromatic segment of Cur within the PLGA segments, signifying the formation of a novel conjugate between the copolymer and Cur.

In the FTIR spectra (Figure 2a), distinct features of the raw materials (Cur and PLGA) and the new conjugate (PLGA-Cur) were analyzed in the range of 4000 to 400 cm^−1^. For pure Cur, characteristic peaks appeared at 3509 cm^−1^ (phenolic –OH stretching vibration), 1628 cm^−1^ (conjugated C=O and C=C stretching), 1519 cm^−1^ (benzene ring), 1273 cm^−1^ (enolic C–O), and 1022 cm^−1^ (C–O–C asymmetric stretching vibration). In contrast, neat PLGA shows typical ester-related bands at 1753 cm^−1^ (C=O stretching), 1185 cm^−1^ and 1080 cm^−1^ (C–O–C stretching), as well as CH_3_ and CH_2_ vibrations in the region 2990–2850 cm^−1^. Upon closer inspection of the FTIR spectra in the region of 2000 to 1000 cm^−1^ (Figure 2b), in the PLGA–Cur conjugate, shifts in the ester carbonyl band from 1753 to 1759 cm^−1^ and of the curcumin carbonyl band from 1628 to 1633 cm^−1^ were observed, together with changes in intensity of the phenolic O–H band, confirming successful covalent conjugation and molecular interactions between curcumin and the PLGA backbone.

**Figure 3:**
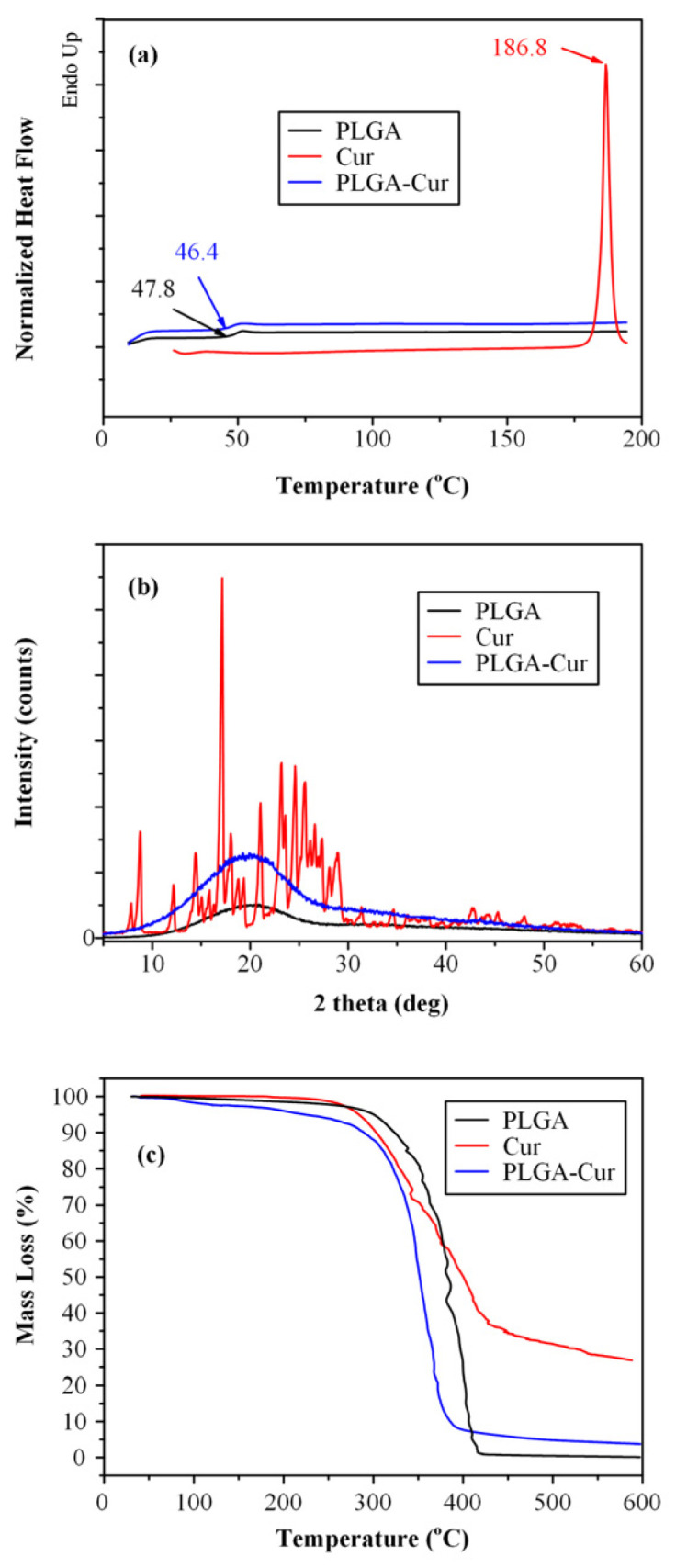
DSC thermograms (**a**) pXRD diffractograms (**b**) TGA thermograms (**c**) of the neat Cur the neat PLGA and the conjugated PLGA-Cur.

Apart from assessing molecular interactions, the physical state of the newly formed conjugate was examined using DSC and pXRD. Figure 3a depicts the DSC thermograms of both the pristine raw materials and the recently synthesized conjugate. Based on the results obtained, PLGA shows a glass transition value (T_g_) equal to 47.8 °C and, as expected, it does not show any melting point endotherms since it is amorphous. As for the pure Cur, it shows an endothermic melting peak at 186.8 °C, indicating that the neat compound is crystalline. In respect to the new conjugate, the obtained DSC results show that the PLGA-Cur conjugate, has only one T_g_ point, located at 46.4 °C which is slightly lower than the neat PLGA, and no endothermic melting peaks, indicating that the new prepared copolymer was also amorphous.

Figure 3b shows the XRD diffractograms of the neat components, as well as the new PLGA-Cur conjugate. Regarding the raw materials, the results showed that Cur is a highly crystalline compound with the major diffraction peaks recorded at 2θ of 12.12, 17.70, 18.81, 22.05, 23.89, 24.98 and 28.01 deg. Conversely, PLGA manifests as an amorphous copolymer, displaying solely a distinct amorphous halo in its pXRD diffractogram. Likewise, the pXRD pattern of the conjugated PLGA-Cur reveals the exclusive presence of an amorphous halo, affirming the development of an amorphous PLGA-Cur copolymer, as corroborated by DSC.

Finally, the thermal stability of the new PLGA-Cur conjugates was evaluated via TGA. Results in Figure 3c showed that the new prepared copolymer, had a slightly higher level of absorbed water in its matrix, as compared to neat PLGA, while it seems to follow the same thermal degradation profile as PLGA, indicating that the addition of Cur did not alter the thermal stability properties of the neat PLGA.

### 3.2. Synthesis and Characterization of the GAL-CDs

Before the preparation of the PLGA-Cur NPs, the API (i.e., GAL) was loaded on the CD-based nanocarrier. This stage was crucial, since an extra layer of protection to the drug is needed, while also the CD-based NPs may facilitate GAL’s controlled release from the final dosage form.

**Figure 4:**
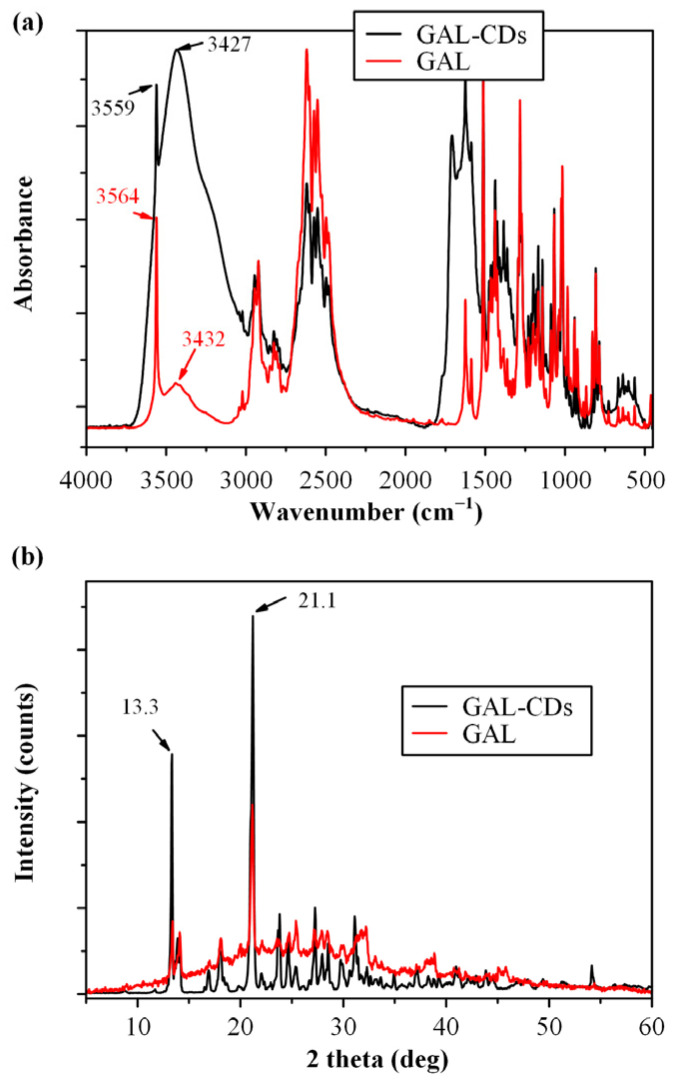
FTIR spectra (**a**) and pXRD diffractograms (**b**) of the neat GAL and the GAL-CDs.

Figure 4a illustrates the FTIR spectra of pure GAL and GAL-CDs. GAL exhibits characteristic peaks at 2834 cm^−1^ (C-H bond stretching vibration), 3432 cm^−1^ (N-H stretching of the quaternary amino group), and 3564 cm^−1^ (-OH vibration). In GAL-CDs, additional peaks from CDs appear at 3260 cm^−1^ (bending vibrations of C-N groups), 1637 cm^−1^, 1371 cm^−1^, and 1023 cm^−1^ originating from oxygen- and nitrogen-containing surface functional groups of the carbon dots. The band at ~1637 cm^−1^ can be attributed to overlapping C=O and C=C stretching vibrations associated with surface amide and carboxyl functionalities of the CDs. Closer inspection reveals shifts and broadening of API-related peaks toward lower wavenumbers, suggesting the formation of molecular interactions, possibly hydrogen bonding and electrostatic interactions, between the drug and CDs. The absence of new absorption bands confirms that galantamine is physically adsorbed or electrostatically associated with the CD surface rather than covalently bonded, which is most likely attributed to (i) the ultrasmall size of the CDs and the absence of internal porosity that limits the available surface area per particle for drug association, (ii) the hydrophilic nature of galantamine, which favors partial desorption during washing and centrifugation, and (iii) the absence of a porous or core–shell structure, meaning that GAL is mainly surface-associated rather than physically entrapped.

In addition to FTIR, the prepared drug loaded CD-NPs were examined via pXRD as well, to evaluate for any changes regarding their physical state. Figure 4b shows the pXRD diffractograms of the neat GAL and the GAL-loaded CDs. Pure GAL exhibited a highly crystalline diffraction pattern, with prominent peaks at 2θ ≈ 13.3° and 21.1°, in agreement with literature reports. After the incorporation upon the carbon dot nanocarrier, all characteristic galantamine diffraction peaks remained clearly detectable, indicating preservation of its crystalline structure. This observation confirms that CD-based incorporation does not induce polymorphic transformation or amorphization of galantamine at this stage, supporting the physical stability of the drug within the CD nanocarrier prior to its incorporation into the polymeric nanoparticles.

### 3.3. Preparation and Characterization of GAL-CDs Loaded into PLGA-Cur NPs

In the final stage of this research work, the newly synthesized PLGA-Cur was used for the preparation of GAL-loaded NPs. Therefore, several variants of these NPs were prepared having: (1) only PLGA and the drug (GAL/PLGA), (2) the new PLGA-Cur copolymer and GAL (GAL/PLGA-Cur), (3) the PLGA and the GAL-loaded CDs (GAL-CD/PLGA), and finally, (4) the PLGA-Cur and the GAL-loaded CDs (GAL-CD/PLGA-Cur).

#### 3.3.1. NP’s Drug Loading, EE and Yield

Table 1 summarizes the results for the drug loading, the EE and the yield results of the prepared nano-formulations. As previously described, all values are reported as mean ± SD (*n* = 3), where *n* refers to technical replicate measurements performed on a single nanoparticle preparation per formulation. Overall, the data indicate that polymer composition and formulation strategy influence galantamine incorporation efficiency, with PLGA–Cur favoring higher mean drug loading when galantamine is directly incorporated into the polymeric matrix, and CD-based intermediate loading leading to lower overall retention.

More specifically, among the formulations containing galantamine directly dispersed within the polymeric matrix, PLGA–Cur nanoparticles exhibited higher mean drug loading and EE values compared with their PLGA counterparts. Specifically, the GAL/PLGA–Cur formulation showed a mean drug loading of 13.65 ± 0.64% and an EE of 38.78 ± 4.50%, whereas GAL/PLGA nanoparticles displayed lower corresponding mean values (9.57 ± 0.73% and 29.04 ± 2.88%, respectively). This trend suggests that conjugation of curcumin to the PLGA backbone may enhance drug–polymer affinity and retention during nanoparticle formation, potentially due to increased molecular interactions or altered polymer chain architecture.

In contrast, formulations in which galantamine was first associated with carbon dots (GAL–CDs) prior to nanoparticle preparation exhibited lower mean drug loading and EE values compared with their non-CD analogs. For example, GAL-CDs/PLGA and GAL-CDs/PLGA–Cur nanoparticles showed mean drug loadings of 6.03 ± 0.90% and 5.33 ± 0.81%, respectively. This reduction is attributed to the two-step loading strategy and is consistent with surface-limited binding and partial drug loss during the subsequent emulsification, solvent evaporation, and washing steps.

Finally, nanoparticle yield followed a comparable trend, with higher mean yields observed for formulations prepared without the intermediate CD-loading step. The GAL/PLGA–Cur formulation exhibited the highest mean yield (89.34 ± 4.01%), while GAL-CD-containing systems showed reduced yields, reflecting the increased complexity and material loss associated with multi-step fabrication processes.

#### 3.3.2. Evaluation of NP’s Morphology, Particle Size and ζ-Potential

**Figure 5:**
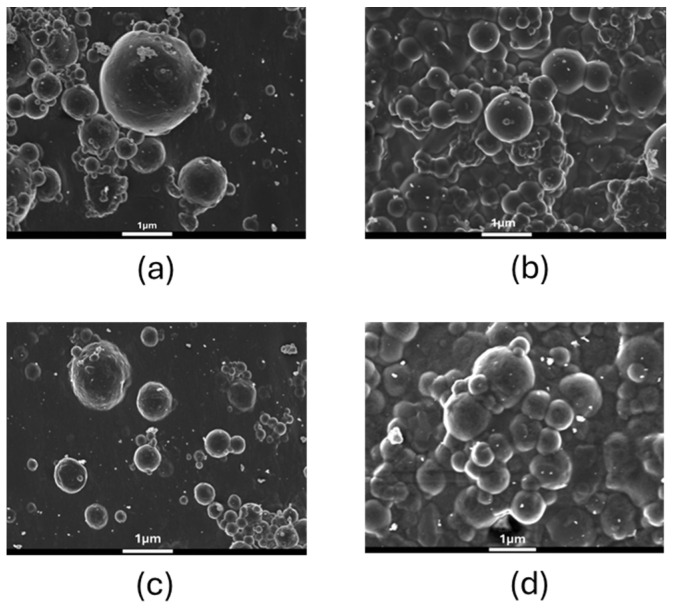
SEM images of the GAL/PLGA (**a**), GAL/PLGA-Cur (**b**), GAL-CDs/PLGA (**c**) and GAL-CDs/PLGA-Cur (**d**).

In a subsequent step, the size and surface morphology of the prepared nanoparticles were examined by dynamic light scattering (DLS) and scanning electron microscopy (SEM), respectively, and the results are presented in Table 1 and Figure 5. All nanoparticle formulations exhibited spherical morphology with smooth or slightly textured surfaces, confirming successful particle formation through the emulsification process.

Table 1 shows the PSD, and the ζ-potential values of all formulated NPs. The resulting data reveal that the particle size of the NPs ranged from 153.7 nm to 256.3 nm, with all samples demonstrating well-dispersed size distributions, as indicated by the PDI values ranging from 0.136 to 0.250. Notably, NPs formulated with the conjugated PLGA-Cur copolymer were larger than those prepared with neat PLGA. This is probably due to the presence of the Cur branch in the PLGA copolymer chain causing steric hindrance phenomena during the solidification process of the NPs, leading to more bulky and consequently larger particles. Additionally, the loading of the API on the CDs resulted in slightly smaller NPs compared to those containing only the neat API. Moreover, Table 1 also provides a summary of the ζ-potential values for all synthesized NPs. In this study, all examined formulations exhibited ζ-potential values above −15 mV, suggesting a diminished propensity for agglomeration and, therefore, an increased likelihood of stabilization. Moreover, to evaluate process reproducibility and particle stability, three independent batches were prepared for each formulation and their particle size (intensity-weighted) was determined (triplicate measurements per batch). The relative standard deviation (RSD) across batches was <2% for all systems, supporting high reproducibility of the preparation process. The results align with the narrow, well-dispersed size distributions reported herein.

In the case of neat PLGA nanoparticles (Figure 5a), some degree of particle agglomeration was evident, resulting in clusters and the appearance of larger-sized aggregates. This behavior is consistent with the higher polydispersity index (PDI = 0.250) determined by DLS, indicating a broader size distribution and lower colloidal stability of the unmodified PLGA system. In contrast, the GAL–PLGA–Cur nanoparticles (Figure 5b) displayed a more uniform dispersion and less aggregation tendency. The presence of curcumin moieties covalently linked to the PLGA backbone is expected to enhance intermolecular compatibility and reduce surface energy, leading to improved colloidal stability. This can be further confirmed by the DLS analysis analyzed above, which revealed a slightly larger mean hydrodynamic diameter (256.3 ± 3.9 nm) compared with neat PLGA NPs (198.0 ± 2.4 nm), but with a lower PDI (0.187), confirming a more homogeneous population of particles.

#### 3.3.3. Evaluation of Drug Physical State

**Figure 6:**
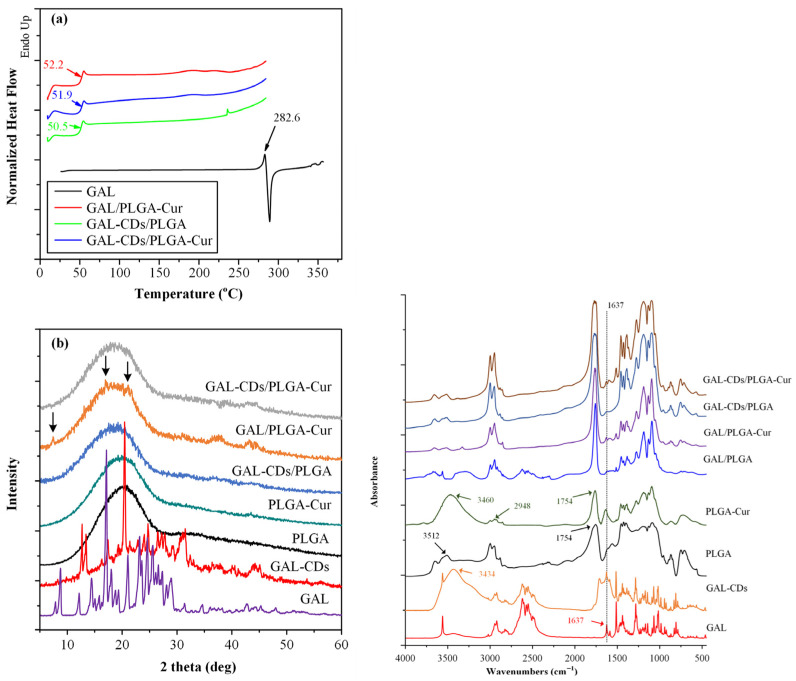
**Left**: DSC thermograms (**a**) and (pXRD diffractograms (**b**)) of the GAL-loaded NPs. (**Right**): FTIR spectra of the GAL-loaded NPs.

Alterations in the physical state of GAL were investigated using DSC, as illustrated in Figure 6a. The results reveal that in all cases, the absence of endothermic peaks corresponding to the melting of the API signifies that GAL was dispersed in an amorphous form within the formulated NPs. This observation indicates that probably a significant change in the physical state of the API occurs during the incorporation of the GAL-CDs into the polymeric NPs. Nonetheless, since in situ amorphization may prevail during the DSC measurements, in order to verify this result, the physical state of the API was evaluated also via PXRD. Figure 6b shows the pXRD diffractograms of the synthesized NPs. There it is seen that, except for the Gal/PLGA-Cur NPs—where partial crystallization of the API is evident and depicted with the black arrows—all other cases showed an amorphous dispersion of the API. This was substantiated by the presence of the broad pXRD halo of the polymeric matrix/carrier in the obtained diffractograms. Intriguingly, this observation was true even for the GAL-CD loaded NPs, despite the initial crystalline state of GAL in this pure nanoformation. This suggests that the incorporation process of the GAL-CD nanoformations into the final polymeric NPs resulted in the total amorphization of the API within the final composite system. This amorphization is hypothesized to occur during s/o/w emulsification and nanoparticle solidification, due to solvent-mediated dispersion, nanoscale confinement, rapid solvent evaporation, and stabilizing interactions between GAL, CDs, and the PLGA–Cur matrix.

#### 3.3.4. Evaluation of Molecular Interactions

In addition to the characterization of the physical state, the prepared systems underwent scrutiny for molecular interactions via FTIR spectroscopy. As illustrated in Figure 6 (right), the FTIR spectra of all components and the NP formulations were examined. The FTIR spectra of GAL/PLGA NPs exhibited dominant peaks attributed to the polymeric matrix-carrier (PLGA), such as those at 1753 cm^−1^ and 1185 cm^−1^ corresponding to the stretching of the C=O ether groups of the copolymer chain. Additional peaks at 1129 and 1455 cm^−1^, corresponding to the C-O-C group and C-H bond of the methyl group, were evident. A closer inspection of the spectra revealed characteristic peaks in GAL as well, such as the sharp peak at 3564 cm^−1^ corresponding to the quaternary amino group of the API. Moreover, several peaks in the spectrum experienced shifts in either lower or higher wavenumbers compared to the neat API; for example, the peaks at 1507 cm^−1^ and 1440 cm^−1^ shifted to 1493 cm^−1^ and 1445 cm^−1,^ respectively, indicative of molecular interactions between the API and the matrix/carrier. Similarly, significant FTIR peak shifts were recorded in the case of GAL/PLGA-Cur, affirming that the presence of Cur in the conjugated copolymer did not alter the potential for the formation of molecular interactions between the drug and the conjugated matrix/carrier.

Concerning the FTIR spectra of the NPs where the drug was initially enclosed within the CDs, in addition to the characteristic FTIR vibrational peaks of the polymer (PLGA or PLGA-Cur) and GAL, numerous peaks corresponding to the CDs were evident. For instance, the peak at 3260 cm^−1^, associated with the bending vibrations of the C-N groups, and the peaks at 1371 cm^−1^ and 1023 cm^−1^, related to the vibrations of CH_2_ and C-N, respectively, were observed. However, upon closer examination of the spectra, it was apparent that certain peaks associated with the API underwent shifts to lower wavenumbers. Notably, the peak at 1637 cm^−1^ (attributed to the vibrations of C-O-C) shifted to 1644 cm^−1^, indicating the presence of molecular interactions when the CDs were introduced into the system.

#### 3.3.5. In Vitro Dissolution Release Results

**Figure 7:**
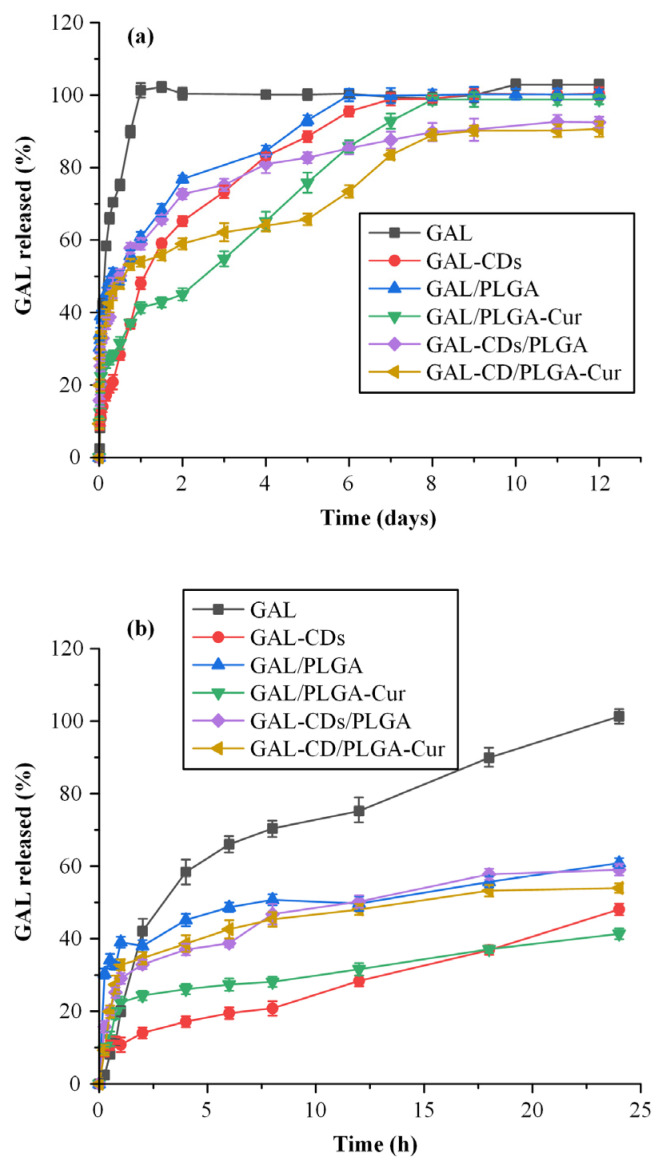
In vitro dissolution profiles of the pure GAL and the prepared NPs up to 12 days (**a**) and in the first 24 h (**b**), (mean ± SD, *n* = 3).

Figure 7a,b depict the in vitro dissolution profiles of all prepared NPs, alongside the profile for neat GAL. Comparisons between formulations were based on release duration, phase-wise release behavior, and kinetic model fitting parameters.

For the pure API, an immediate release profile was observed, with 100% of the drug released within the initial 24 h, in line with sink conditions prevailing throughout the dissolution testing period. In contrast, the GAL-CDs NPs showcased a controlled, three-stage release of the API, suggesting a distinct and regulated dissolution pattern facilitated by the presence of CDs, in comparison to the unmodified GAL. Specifically, from the begging of the dissolution process and up to 8 h, an immediate release of the drug from the adsorbed nanostructure is noted, potentially attributable to the quantity of GAL deposited on the outer surface of the CDs. This burst release phase is succeeded by a second stage, extending from 8 h to ~1.5 days, characterized by a sustained release profile. Then, a third stage appears, where the API is still being released in a controlled manner up to until ~day 7, at which point the release from the nanostructure attains 100%, in which, however, the rate of dissolution seems to be faster as compared to the previous sustained release phase. This multi-phase release behavior seems to be controlled by the physicochemical characteristics of the embedded CDs, including their surface chemistry and electrostatic properties (Section 2.4.1).

Regarding the remaining NPs that use either PLGA or PLGA-Cur as a matrix/carrier, three stage dissolution profiles were obtained in all cases, with the exceptions of GAL-CDs/PLGA-Cur. Specifically, all release profiles exhibited distinct phases: (i) an initial burst release stage lasting around 3 h (phase I), (ii) a sustained drug release phase persisting for approximately 2 days (phase II), and (iii) a more rapid sustained release phase continuing until roughly 8–10 days (phase III). The initial burst release (phase I) is attributed to GAL located on the surface, while the sustained drug release phases (phases II and III) are linked to the swelling (i.e., gel formation) and biodegradation (via erosion) properties of the NPs’ matrix/carriers. Specifically, for NPs containing pure GAL, a triphasic release profile was consistently observed across both the unmodified (PLGA) and modified (PLGA-Cur) polymers. The slower release from PLGA–Cur-based nanoparticles may be attributed to curcumin conjugation, which increases matrix hydrophobicity and drug–polymer interactions, thereby limiting water penetration, polymer relaxation, and galantamine diffusion.

Specifically, for both polymer variants, an immediate release of the drug was evident in the initial 8 h, likely due to the GAL presence on the exterior, or near the surface, of the NPs, reaching approximating 40% and 25% for PLGA and PLGA-Cur based NPs, respectively. Hence, it seems that, at least when the pure GAL (and not the GAL-CDs nanoformations) is embedded into the NPs, the presence of the Cur modification in the PLGA matrix/carrier was able to suppress drug’s initial burst effect. This immediate release phase was succeeded by a second stage, manifesting a controlled release of the drug up to ~2 days, achieving 80% and 45% for the PLGA and PLGA-Cur copolymers, respectively. Finally, a third controlled release stage was seen in both formulations, in which the drug release was continued up to 6 and 8 days for PLGA and PLGA-Cur, respectively. Hence, in all dissolution phases, it seems that the presence of Cur results into slower GAL release profiles, a finding that can be attributed to the presence of enhanced molecular interactions between the drug and the Cur moieties in the polymer, or to the stereochemical hindrance introduced by the Cur segments in the conjugated PLGA-Cur copolymer.

For the NPs synthesized with GAL-CDs, again a three-stage release pattern was noted for the case of PLGA, whereas a four-stage pattern was seen for the new conjugated PLGA-Cur. In the initial stage, which was common in both cases, a similar initial burst release was observed lasting up to ~2 h. Then, a common sustain release phase was observed, up until ~2 days, releasing approximately 80% and 60% of the API for the PLGA and the PLGA-Cur NPs, respectively. Subsequently, a divergence in the release patterns of the two copolymers is observed. For the PLGA-based NPs, a third sustained release stage is seen, extending up to ~11 days, where all the remaining API was released from the formulations, whereas for the PLGA-Cur NPs, there seem to be a lag phase starting from ~2 days up to 5 days, where almost no API is released from the NPs, followed by a fourth, and final, sustained release phase, where up to ~90% of the API is released until day 8. The four-phase release profile of the GAL–CD/PLGA–Cur formulation is hypothesized to arise from surface-associated drug release, followed by diffusion from GAL confined within CD nanodomains, a transient lag phase related to polymer hydration and curcumin-induced diffusion barriers, and a final sustained release governed by matrix swelling and erosion.

Compared to our previously reported galantamine nanoformulations [23,31], in the present system, galantamine loading values fall within a comparable range (10–20%), while the incorporation of carbon dots within a PLGA–Cur matrix enables prolonged and more finely modulated release (up to 10–12 days) through the combined effects of nanoscale drug confinement, polymer degradation, and curcumin-induced hydrophobic diffusion barriers. Therefore, unlike chitosan-based carriers, whose performance is strongly influenced by environmental pH and ionic strength, or other systems where the release is governed only by polymer swelling and diffusion-controlled mechanisms, the proposed hybrid PLGA–Cur/CD platform represents a complementary strategy for long-acting intranasal galantamine delivery, as it combines the improved physicochemical stability and favorable degradation kinetics of the PLGA-based matrix with the observed multi-phase release behavior arising from the CDs and curcumin conjugation.

To further explore the mechanism of galantamine (GAL) release from the prepared nanoparticles, the obtained dissolution profiles were fitted to various kinetic models (Equations (4)–(8)). Fitting was performed separately for each release phase (Phase II and Phase III), except for the initial burst phase (Phase I), where the mechanism is clearly dominated by solubilization and diffusion of surface-associated drug molecules. The corresponding goodness-of-fit results, summarized in Table 2, were assessed based on the correlation coefficient (R^2^) and the kinetic constants (k) for each model. Model selection was based on phase-wise fitting of the sustained release regions and comparison of correlation coefficients (R^2^), an approach commonly adopted for the analysis of multiphasic release behavior in polymeric nanoparticle delivery systems. The data showed excellent linearity in the zero-order model, suggesting a balanced contribution of diffusion and matrix erosion processes in governing the sustained release behavior. Once diffusion was established as the primary mechanism, the Korsmeyer–Peppas model was applied to further clarify the nature of the diffusion process. According to this model, the release exponent (*n*) provides insight into the underlying transport mechanism. As shown in Table 2, the obtained *n* values were ≥0.5, indicating that galantamine release followed an abnormal (non-Fickian) diffusion mechanism [29]. This implies that the release was not controlled solely by molecular diffusion through the hydrated polymeric network but was also influenced by polymer swelling and gradual matrix erosion. As the PLGA-based nanoparticles absorbed water, polymer chains progressively relaxed and partially dissolved, resulting in a simultaneous contribution of diffusion and erosion to the overall release process. Among the studied formulations, the GAL–CD/PLGA–Cur hybrid nanoparticles exhibited the lowest release constant (k) and the most prolonged release duration, consistent with their denser matrix structure and stronger drug–carrier interactions due to the integration of curcumin and carbon dots. Collectively, these findings demonstrate that the proposed hybridization strategy effectively modulates the diffusion–erosion balance, yielding a controlled, non-Fickian sustained release profile characteristic of complex PLGA-based nanocarriers.

## 4. Conclusions

In this investigation, various new hybrid GAL nanoformulations were evaluated for the first time. In these systems, the API was loaded on CDs and then embedded into PLGA-Cur modified NPs, in an attempt to improve GAL’s therapeutic outcomes against AD, by dismantling amyloid plaques with Cur, while ensuring superior dissolution characteristics and extra protection of the drug with the use of CDs. The findings indicate that the incorporation of Cur into PLGA and the utilization of CD exert substantial influence on the physicochemical attributes and the in vitro dissolution performance of the newly formulated GAL-loaded NPs. For example, the use of the PLGA-Cur copolymer led to higher drug loadings and slightly larger NP sizes compared to the neat PLGA formulations. In addition, the formation of significant molecular interactions between the API and the two matrix/carriers was unraveled, thus providing an insight into the structural synergy phenomena occurring within the prepared nano-formulations. Finally, the in vitro dissolution studies confirmed a sustained release pattern, supporting the system’s potential to maintain prolonged GAL availability. However, these results represent a proof of concept, and no direct therapeutic or in vivo conclusions can yet be drawn. The present work therefore constitutes an initial step toward the development of hybrid PLGA–Cur/CD nanoparticles for intranasal GAL delivery. Future investigations will focus on in vitro and ex vivo studies of nasal permeability, mucosal retention, and cytocompatibility, followed by in vivo pharmacokinetic and neurobehavioral evaluations to validate the efficacy and suitability of the proposed delivery route.

## Figures and Tables

**Table 1:** Summary of drug loading, EE, NP yield results and NPs’ PSD, PDI and ζ-potential results measured via DLS (mean ± SD, *n* = 3).

Sample	Drug Loading (%)	EE (%)	NPs’ Yield (%)	Mean Particle Size (nm)	RSD (%)	PDI	ζ-Potential (mV)
GAL/PLGA	9.57 ± 0.73	29.04 ± 2.88	78.71 ± 3.15	198.0 ± 2.35	1.2	0.250	−27.4 ± 0.9
GAL/PLGA-Cur	13.65 ± 0.64	38.78 ± 4.50	89.34 ± 4.01	256.3 ± 3.89	1.5	0.136	−18.2 ± 0.7
GAL-CDs/PLGA	6.03 ± 0.90	16.70 ± 1.34	43.30 ± 2.72	153.7 ± 1.77	1.2	0.144	−22.6 ± 1.1
GAL-CDs/PLGA-Cur	5.33 ± 0.81	11.65 ± 1.02	57.56 ± 3.19	220.5 ± 2.01	0.9	0.143	−24.1 ± 1.4

**Table 2:** Dissolution data model fitting results for the employed drug release kinetic models.

Release Model	NPs’ Formulations							
	GAL/PLGA		GAL/PLGA-Cur		GAL-CDs/PLGA		GAL-CDs/PLGA-Cur	
	R^2^	k	R^2^	k	R^2^	k	R^2^	k
Phase II								
Zero order	0.993	17.6 d^−1^	0.950	9.73 d^−1^	0.970	14.89 d^−1^	0.978	7.45 d^−1^
First order	0.315	0.80 d^−1^	0.830	0.33 d^−1^	0.691	0.24 d^−1^	0.315	0.80 d^−1^
Higuchi	0.464	36.73 d^−1/2^	0.926	31.51 d^−1/2^	0.530	33.10 d^−1/2^	0.498	30.48 d^−1/2^
Hixson-Crowell	0.378	0.13 d^−1^	0.800	0.08 d^−1^	0.386	0.13 d^−1^	0.129	0.11 d^−1^
Korsmeyer-Peppas	0.984	64.80 d^−^*^n^* (*n* = 0.196)	0.971	41.71 d^−^*^n^* (*n* = 0.352)	0.989	56.61 d^−^*^n^* (*n* = 0.212)	0.958	52.49 d^−^*^n^* (*n* = 0.206)
Phase III or IV								
Zero order	0.968	5.80 d^−1^	0.981	8.40 d^−1^	0.964	2.63 d^−1^	0.994	8.90 d^−1^
First order	0.612	1.36 d^−1^	0.829	3.28 d^−1^	0.691	1.22 d^−1^	0.534	0.12 d^−1^
Higuchi	0.388	36.80 d^−1/2^	0.925	31.51 d^−1/2^	0.531	0.33 d^−1/2^	0.508	30.25 d^−1/2^
Hixson-Crowell	0.287	0.13 d^−1^	0.800	0.08 d^−1^	0.386	0.12 d^−1^	0.206	0.12 d^−1^
Korsmeyer-Peppas	0.984	64.52 d^−^*^n^* (*n* = 0.194)	0.970	41.71 d^−^*^n^* (*n* = 0.352)	0.989	56.61 d^−^*^n^* (*n* = 0.212)	0.969	53.46 d^−^*^n^* (*n* = 0.207)

## Data Availability

The original contributions presented in this study are included in the article. Further inquiries can be directed to the corresponding author.
